# The efficacy of neoadjuvant chemohormonal therapy in combination with radical prostatectomy for locally advanced prostate cancer: a systematic review and meta-analysis

**DOI:** 10.3389/fonc.2026.1693222

**Published:** 2026-04-20

**Authors:** Hua Luo, Xiaobo Wang, Chao He

**Affiliations:** 1 Department of Urology, Deyang Hospital of Sichuan Provincial People’s Hospital, Deyang, China; 2 Department of Urology, Deyang Second People’s Hospital, Sichuan, Deyang, China

**Keywords:** androgen deprivation therapy (ADT), neoadjuvant chemohormonal therapy, prostate cancer, radical prostatectomy, randomized controlled trial

## Abstract

**Objective:**

Locally advanced prostate cancer (clinical stage T3 or higher) causes significant harm to patients, including decreased quality of life, high mortality rates, and economic burden. Current multimodal management for locally advanced prostate cancer often includes radiotherapy combined with long-term androgen deprivation therapy (ADT), with radical prostatectomy (RP) reserved for selected patients. However, RP alone or with ADT has limitations such as high biochemical recurrence rates and high positive surgical margin (PSM) rates. Therefore, this study aims to explore new effective treatment strategies.

**Methods:**

In accordance with the Preferred Reporting Items for Systematic Reviews and Meta-Analyses (PRISMA) guidelines, we systematically searched PubMed, Embase, Web of Science and Cochrane databases up of August 19, 2025. We analyzed data from 10 studies. The main outcomes assessed included biochemical progression-free survival (BPFS), PSM rate, overall survival (OS) and metastasis-free survival (MFS). We pooled risk ratios (RR) with 95% confidence intervals (CI) using fixed- or random-effects models.

**Results:**

The results show that compared with RP alone, the combination of NCHT and RP significantly improved BPFS (RR = 0.60, 95% CI: 0.41-0.87, P = 0.008), despite significant heterogeneity (I² = 90%). In RCT subgroup analysis, the benefit remained significant (RR = 0.73, 95% CI: 0.62–0.86; P = 0.0002; I² = 0%). The NCHT+RP group also showed a significant reduction in PSM rate (RR = 0.49, 95% CI: 0.37-0.65, P < 0.00001). For OS, the pooled analysis showed an improvement (RR = 0.45, 95% CI: 0.24-0.84, P = 0.01); however, this effect was entirely driven by non-randomized studies (RR = 0.55, 95% CI: 0.31–0.99; P = 0.04), while the randomized controlled trials (RCT) subgroup did not confirm a significant benefit (RR = 0.43, 95% CI: 0.10-1.88, P = 0.26). No significant improvement was observed in MFS (RR = 0.82, 95% CI: 0.45-1.49, P = 0.51), although the single large RCT (Eastham et al.) favored NCHT+RP (RR = 0.78, 95% CI: 0.63–0.98; P = 0.03).

**Conclusion:**

In selected patients with locally advanced prostate cancer, NCHT combined with RP may improve BPFS and reduce PSM rates, with the BPFS benefit confirmed in the RCT subgroup. However, the apparent OS benefit was not observed in RCTs and likely reflects bias in observational studies. According to GRADE, the certainty of evidence for survival outcomes is very low. These positive findings should be interpreted with caution, and adequately powered RCT with modern agents are needed.

## Introduction

Prostate cancer (PCa) remains one of the most prevalent malignancies among men worldwide, with locally advanced disease (clinical stage T3 or higher) posing significant therapeutic challenges (1). These patients often experience reduced quality of life, high mortality rates, and substantial economic burdens due to costly treatments and long-term care (2–4). Current multimodal management for locally advanced PCa most commonly involves radiotherapy combined with long-term androgen deprivation therapy (ADT), with radical prostatectomy (RP) considered for selected patients (5). These approaches are associated with persistent limitations, such as high rates of biochemical recurrence and positive surgical margins (PSM), underscoring the need for more effective treatment strategies.

However, the landscape of neoadjuvant therapy for high-risk prostate cancer is rapidly evolving. Contemporary trials are increasingly focused on intensified androgen receptor pathway inhibitors (ARPIs; e.g., enzalutamide, apalutamide, darolutamide) with or without ADT, which have demonstrated superior pathologic complete response (pCR) rates compared to historical docetaxel-based chemohormonal therapy in early-phase studies (6). The largest randomized controlled trial to date evaluating neoadjuvant docetaxel-based neoadjuvant chemohormonal therapy (NCHT) (CALGB 90203) did not meet its primary endpoint of 3-year biochemical progression-free survival, and current National Comprehensive Cancer Network (NCCN) guidelines do not recommend neoadjuvant ADT or chemotherapy prior to radical prostatectomy outside of clinical trials (7). Therefore, while our meta-analysis focuses on the available evidence for NCHT+RP, its findings must be interpreted within this evolving therapeutic context.

NCHT, which combines ADT with chemotherapy prior to RP, has emerged as a promising approach to downstage tumors and improve surgical outcomes. While some studies suggest potential benefits in biochemical progression-free survival (BPFS), evidence regarding its impact on overall survival (OS) and metastasis-free survival (MFS) remains inconclusive (8, 9). Previous systematic reviews have reported mixed results, partly due to variations in study design, patient populations, and treatment protocols (10).

Given these inconsistent findings and to provide a more comprehensive evaluation, we conducted a systematic review and meta-analysis of both randomized controlled trials (RCTs) and comparative cohort studies. This study aims to synthesize current evidence on the efficacy of NCHT combined with RP versus RP alone, focusing on key oncological outcomes including BPFS, OS, PSM, and MFS. Our findings seek to inform clinical decision-making and highlight directions for future research.

## Materials and methods

### Protocol and guidance

This systematic review and meta-analysis was conducted in accordance with the Preferred Reporting Items for Systematic Reviews and Meta-Analyses (PRISMA) guidelines (**Supplementary Table 1**) (11). Our protocol has been registered with the International Prospective Register of Systematic Reviews (PROSPERO, CRD420251123186).

### Data sources, search strategy and definitions

A thorough review of existing literature was conducted up to August 19, 2025, utilizing the PubMed, Embase, Web of Science, and Cochrane databases. Our search incorporated terms such as neoadjuvant chemohormonal therapy, radical prostatectomy, prostate cancer, locally advanced prostate cancer, and oncological outcomes. To enhance our search, we also included synonyms related to these key concepts found in titles, abstracts, and keywords across the databases. Detailed search strategies for each database can be found in **Supplementary Table 2**.

### Study selection: inclusion and exclusion criteria

Detailed inclusion criteria were defined according to the PICOS framework (**Table 1**). Inclusion criteria were: 1) Population: enrolled patients with histologically confirmed locally advanced prostate cancer (clinical stage T3 or higher); 2) Intervention: administered NCHT neoadjuvant chemohormonal therapy (any regimen of ADT combined with chemotherapy) prior to RP; 3) Comparator: compared the intervention with RP alone; 4) Outcomes: reported data on at least one of BPFS, OS, PSM rate, or MFS; 5) Study Design: RCTs or comparative cohort studies. Studies must include a clear definition of the intervention and comparator groups. We excluded studies without a control group, non-comparative designs (e.g., case reports, editorials, conference abstracts), and those lacking relevant outcome data.

**Table 1:** PICOS eligibility criteria for inclusion of studies in the meta-analysis.

P (Population)	I (Intervention)	C (Comparator)	O (Outcomes)	S (Study Design)
Patients with locally advanced prostate cancer (clinical stage T3 or higher)	Neoadjuvant chemohormonal therapy (NCHT), consisting of androgen deprivation therapy (ADT) combined with chemotherapy, administered prior to radical prostatectomy (RP)	Radical prostatectomy (RP) alone	Biochemical progression-free survival (BPFS) Positive surgical margin (PSM) rate Overall survival (OS) Metastasis-free survival (MFS)	Randomized controlled trials (RCTs) Comparative cohort studies

### Data items and extraction process

**Figure 1:**
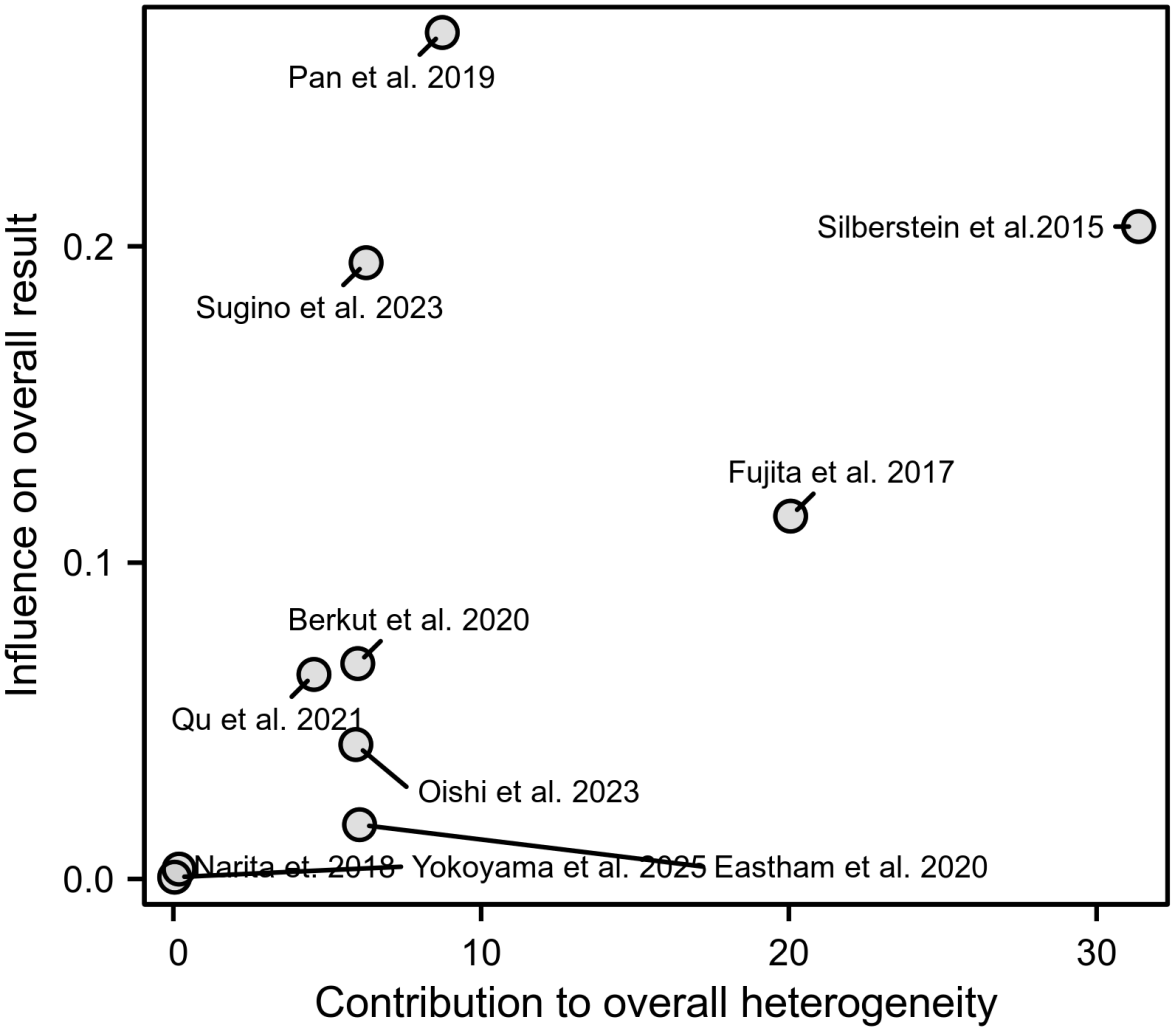
Flow diagram of study selection according to PRISMA guidelines.

The flow chart illustrating the selection process of studies and the rationale for exclusions is depicted in **Figure 1**. Initially, duplicates were eliminated, followed by a review of titles and abstracts to assess their relevance. The full-texts of the articles were then scrutinized for eligibility based on previously established criteria. A standardized, pre-piloted data extraction form was used. Extracted items included: study identifiers (first author, year, country); study design and methodology; participant characteristics; intervention details; comparator details; outcome definitions and measurement timepoints; and results for each outcome. For time-to-event outcomes, we sought to extract hazard ratios (HRs) from multivariate analyses. If these were not reported, we extracted event numbers at the longest available follow-up to calculate risk ratios (RRs), as described in the statistical analysis section. All extraction was performed independently by two authors (X.B.W, C.H) with conflicts resolved by consensus or third-author (H.L) adjudication.

### Risk of bias assessment

The Cochrane ROB 2 tool was utilized to evaluate the bias risk in the RCTs we included (12). These assessments were derived from the information presented in each study, categorizing the bias risk as “Low,” “High,” or “ some concern”. Each study was assigned a classification of low-risk, some concern or high-risk based on these evaluations, with explanations provided for each rating (**Table 2**). The methodological quality of the six included retrospective cohort studies was evaluated using the Newcastle-Ottawa Scale (NOS) (**Table 3**) (13). Two authors (X.B.W, C.H) conducted independent evaluations of the study quality, and any disagreements were addressed with the assistance of a third author (H.L).

**Table 2:** Risk of bias assessment using the Cochrane RoB 2 tool across included studies.

Study	D1	D2	D3	D4	D5	Overall
Eastham et al, 2020 (14)	Low	Low	Low	Low	High	High
Narita et al, 2018 (23)	Low	Low	Low	Low	Low	Low
Qu et al, 2021 (17)	Low	Low	Low	Low	Low	Low
Berkut et al, 2020 (16)	Low	Low	Low	Low	Low	Low

**Table 3:** Quality assessment of non-randomized studies using the Newcastle–Ottawa Scale (NOS).

Study, country	Selection (4)				Comparability (2)		Outcome (3)			Total score
	Representativeness of the exposed cohort	Selection of the non-exposed cohort	Ascertainment of exposure	Demonstration that outcome of interest was not present at start of study	Age comparability	Additional comparability	Assessment of Outcome	Enough follow-up	Adequacy of follow up of Cohorts	
Fujita et al., 2017 (19), Japan	1	1	1	1	1	1	1	1	1	9
Silberstein et al., 2015 (15), USA	0	1	1	1	1	0	1	1	1	7
Yokoyama et al., 2025 (20), Japan	1	1	1	1	1	1	1	1	1	9
Sugino et al., 2023 (21), Japan	1	1	1	1	1	1	1	1	1	9
Oishi et al., 2023 (22), Japan	0	0	0	1	0	0	1	1	0	3
Pan et al., 2019 (18), China	0	1	1	1	1	1	1	1	0	7

### Evidence quality assessment

This research utilized the GRADE (Grading of Recommendations Assessment, Development and Evaluation) framework to assess the quality of evidence concerning the primary outcome measures. The evaluation through GRADE was influenced by five factors that could lead to downgrading: bias in studies, inconsistency of results, indirectness, lack of precision and publication bias. Each outcome’s evidence was categorized into one of four levels: “high”, “medium”, “low” or “very low”. The assessment was conducted independently by two researchers, and the findings were compiled using GRADEpro GDT software (https://gradepro.org). If any disagreements arose, a third researcher was brought in to provide a resolution and facilitate consensus.

### Statistical analysis

The analysis of data was performed utilizing Review Manager 5.3 software provided by the Cochrane Collaboration. For time-to-event outcomes, (BPFS, OS, MFS), we planned to extract hazard ratios (HRs) with 95% confidence intervals from multivariate analyses as the preferred effect measure. However, several included studies did not report HRs or provide Kaplan–Meier curves with sufficient detail to allow extraction of individual patient data or calculation of HRs using established methods. Therefore, as a pragmatic approach, we pooled risk ratios (RRs) at the longest available follow-up for each study. We acknowledge that this approach does not account for time-to-event nature of the data, censoring, or differential follow-up durations, and may either underestimate or overestimate treatment effects. This limitation is addressed in the discussion. Statistical heterogeneity was assessed using the Cochran Q test and I² statistic, with I² ≥ 50% indicating substantial heterogeneity. We explored potential sources of heterogeneity through prespecified subgroup analyses (by study design, geographic region, and chemotherapy regimen) and sensitivity analyses using the leave-one-out method. Given the small number of RCTs for OS and MFS, subgroup results should be interpreted with caution due to limited statistical power. Publication bias was assessed using funnel plots, Egger’s test, and trim-and-fill analysis, although the power of these tests is limited with fewer than 10 studies.

## Results

### Study selection and characteristics

A systematic search across PubMed, EMBASE, Web of Science, and Cochrane yielded 2,075 records. After removing 635 duplicates, 1,440 studies underwent title and abstract screening, excluding 1,332 records. Of 108 full-text articles assessed, 95 were excluded due to: absence of a control group (n = 15), incomplete data (n = 8), irrelevant outcomes (n = 11), conference abstracts (n = 8), or non-original research (n = 53). Ultimately, 13 studies were included for qualitative synthesis, and 10 studies (comprising 4 randomized controlled trials and 6 retrospective cohort studies, with a total of 2,761 patients) were eligible for quantitative meta-analysis (**Figure 1**). Three studies were excluded from quantitative synthesis due to unmergeable data. Ultimately, the final selection consisted of four RCTs and six retrospective cohorts, with two conducted in USA (14, 15), one in Russia (16), two in China (17, 18), and five in Japan (19–23). Ten studies compared NCHT plus RP versus RP alone (**Table 4**).

**Table 4:** Baseline characteristics and interventions included in the meta-analysis.

Author, Year	Country	Study design	Interventions	Sample size		Age (years)		Follow−up (months)	
				NCHT+RP	RP	NCHT+RP	RP	NCHT+RP	RP
Eastham et al., 2020 (14)	USA	RCT	Docetaxel + androgen deprivation + RP vs. RP alone	391	397	62 (IQR:40 - 78)	63 (IQR:33 - 84)	73.2 (IQR:0 -145.2)	73.2 (IQR:0 -145.2)
Narita et al., 2018 (23)	Japan	RCT	Docetaxel + estramustine + androgen blockade + RP vs. RP alone	56	56	71 (IQR:69 - 75)	72 (IQR:69 - 75)	14.0 (IQR:6.0 -25.5)	25.6 (IQR:11.3 -47.2)
Qu et al., 2021 (17)	China	RCT	Docetaxel + androgen deprivation + RP vs. RP alone	42	54	64 (IQR:57 – 75)	67 (IQR:60 – 75)	NA	NA
Berkut et al., 2020 (16)	Russia	RCT	Docetaxel + androgen deprivation + RP vs. RP alone	36	35	62.88 ± 7.37	63.77 ± 7.23	32.78 ± 16.49	42.69 ± 22.34
Fujita et al., 2017 (19)	Japan	Retrospective	Estramustine + androgen deprivation + RP vs. RP alone	436	177	68 (IQR: 64 –7 2)	68 (IQR:64 – 71)	48.8 (IQR:28.4 –77.8)	111 (IQR:83.7 –138)
Silberstein et al., 2015 (15)	USA	Retrospective	Paclitaxel + carboplatin + estramustine + RP vs. RP alone	34	123	56.0 (IQR:51.0 - 61.0)	60.8 (IQR:55.5 -64.9)	157.2	147.6
Sugino et al., 2023 (21)	Japan	Retrospective	Androgen deprivation + tegafur-Uracil + RP vs. RP alone	101	126	70.0 (IQR:67.0 – 73.0)	72.0 (IQR:68.0 –74.0)	17.5 (IQR:6.2 –35.2)	12.(IQR:5.0 –29.0)
Oishi et al., 2023 (22)	Japan	Retrospective	Estramustine+ androgen deprivation + RP vs. RP alone	302	150	68 (IQR:65 - 72)	70 IQR:(66 - 72)	80 (IQR:56 – 98)	41 (IQR:27 – 58)
Yokoyama et al., 2025 (20)	Japan	Retrospective	Androgen deprivation + tegafur-Uracil + RP vs. RP alone	139	139	72 (IQR:69 – 75)	71 (IQR:69 – 75)	14.0 (IQR:6.0 –25.5)	25.6 (IQR:11.3 –47.2)
Pan et al., 2019 (18)	China	Retrospective	Docetaxel + androgen deprivation + RP vs. RP alone	60	44	65 (IQR:46 - 78)	69 (IQR:57 - 78)	12.5	22.8

**Figure 2:**
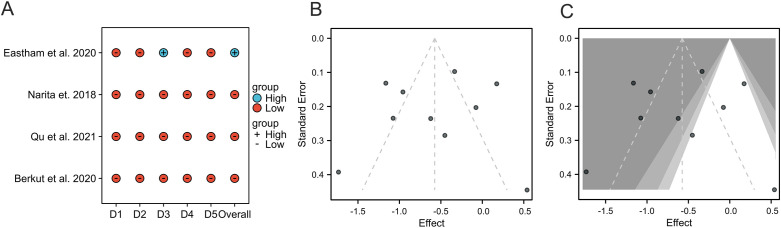
Forest plots for BPFS. **(A)** Overall analysis of NCHT+RP vs. RP alone. **(B)** Subgroup analysis of RCTs. **(C)** Subgroup analysis of non-randomized studies.

This meta-analysis encompassed nine studies that assessed the impact of NCHT in conjunction with RP on BPFS. The NCHT+RP group demonstrated significantly improved BPFS versus RP alone in the overall analysis (RR = 0.60, 95% CI: 0.41– 0.87; P = 0.008; I^2^ = 90%) (**Figure 2A**). However, due to substantial heterogeneity and inclusion of observational studies, the RCT-only subgroup provides the most reliable estimate (RR = 0.73, 95% 95% CI: 0.62 - 0.86; P = 0.0002; I^2^ = 0%) (**Figure 2B**). The non-RCTs showed benefit persisted but with high heterogeneity (RR = 0.53, 95% CI: 0.29 – 0.97; P = 0.04; I^2^ = 93%) (**Figure 2C**).

**Figure 3:**
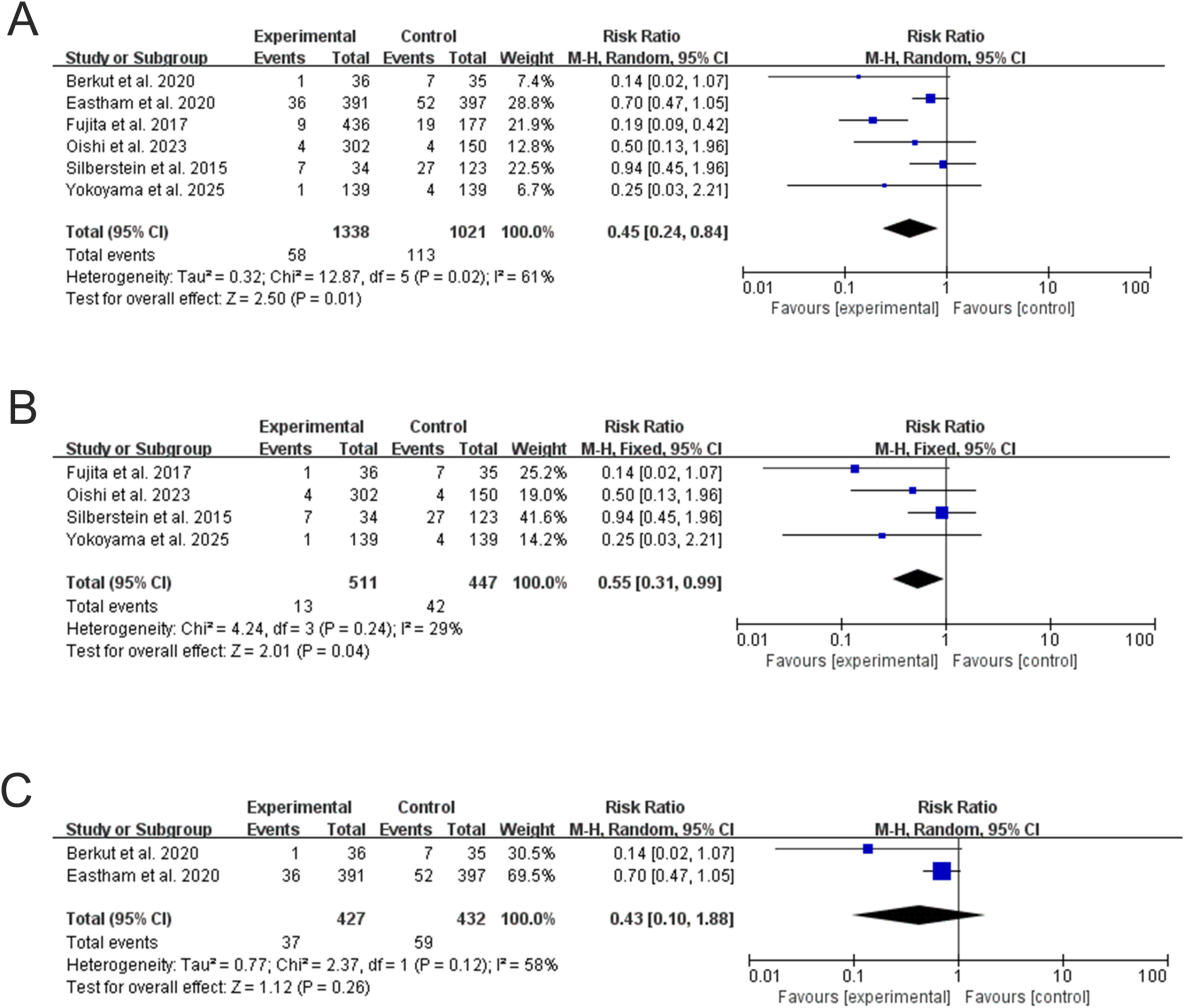
Forest plots for OS. **(A)** Overall analysis of NCHT+RP vs. RP alone. **(B)** Subgroup analysis of non-randomized studies. **(C)** Subgroup analysis of RCTs.

We conducted an analysis of overall survival (OS). For overall survival (OS), the RCT subgroup did not demonstrate a statistically significant benefit with NCHT+RP (RR = 0.43, 95% CI: 0.10–1.88; P = 0.26; I² = 53%) (**Figure 3C**), based on three RCTs with limited events and low statistical power. In contrast, the non-randomized studies showed a significant improvement (RR = 0.55, 95% CI: 0.31–0.99; P = 0.04; I² = 36%) (**Figure 3B**), but these estimates are susceptible to selection bias. The overall pooled analysis, combining both designs, yielded a nominally significant result (RR = 0.45, 95% CI: 0.24–0.84; P = 0.01; I² = 61%) (**Figure 3A**); however, this finding is driven entirely by the non-randomized studies and should be interpreted with caution given the lack of confirmation in RCTs and the methodological limitations of pooling disparate designs.

**Figure 4:**
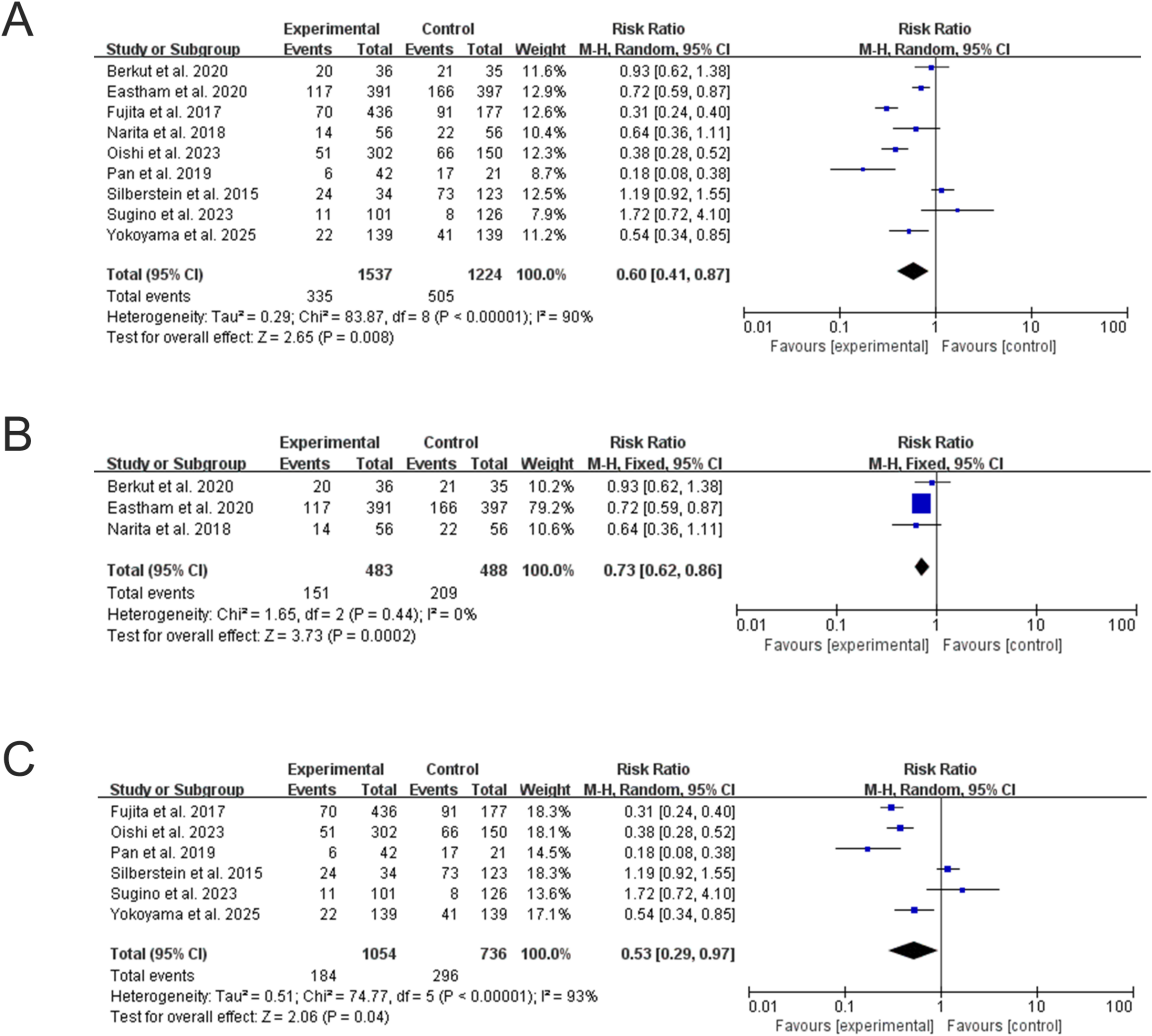
Forest plots for PSM rates. **(A)** Overall analysis. **(B)** Subgroup analysis of RCTs. **(C)** Subgroup analysis of non-randomized studies.

For positive surgical margin (PSM) rates, the RCT subgroup confirmed a significant reduction with NCHT+RP (RR = 0.52, 95% CI: 0.32–0.84; P = 0.007; I² = 79%) (**Figure 4B**). The non-randomized studies also showed a significant benefit (RR = 0.45, 95% CI: 0.35–0.58; P < 0.00001; I² = 47%) (**Figure 4C**), with lower heterogeneity. The overall pooled analysis, consistent with the subgroups, demonstrated a significant reduction in PSM (RR = 0.49, 95% CI: 0.37–0.65; P < 0.00001; I² = 65%) (**Figure 4A**), although moderate heterogeneity remained, largely attributable to differences in study populations and chemotherapy regimens.

**Figure 5:**
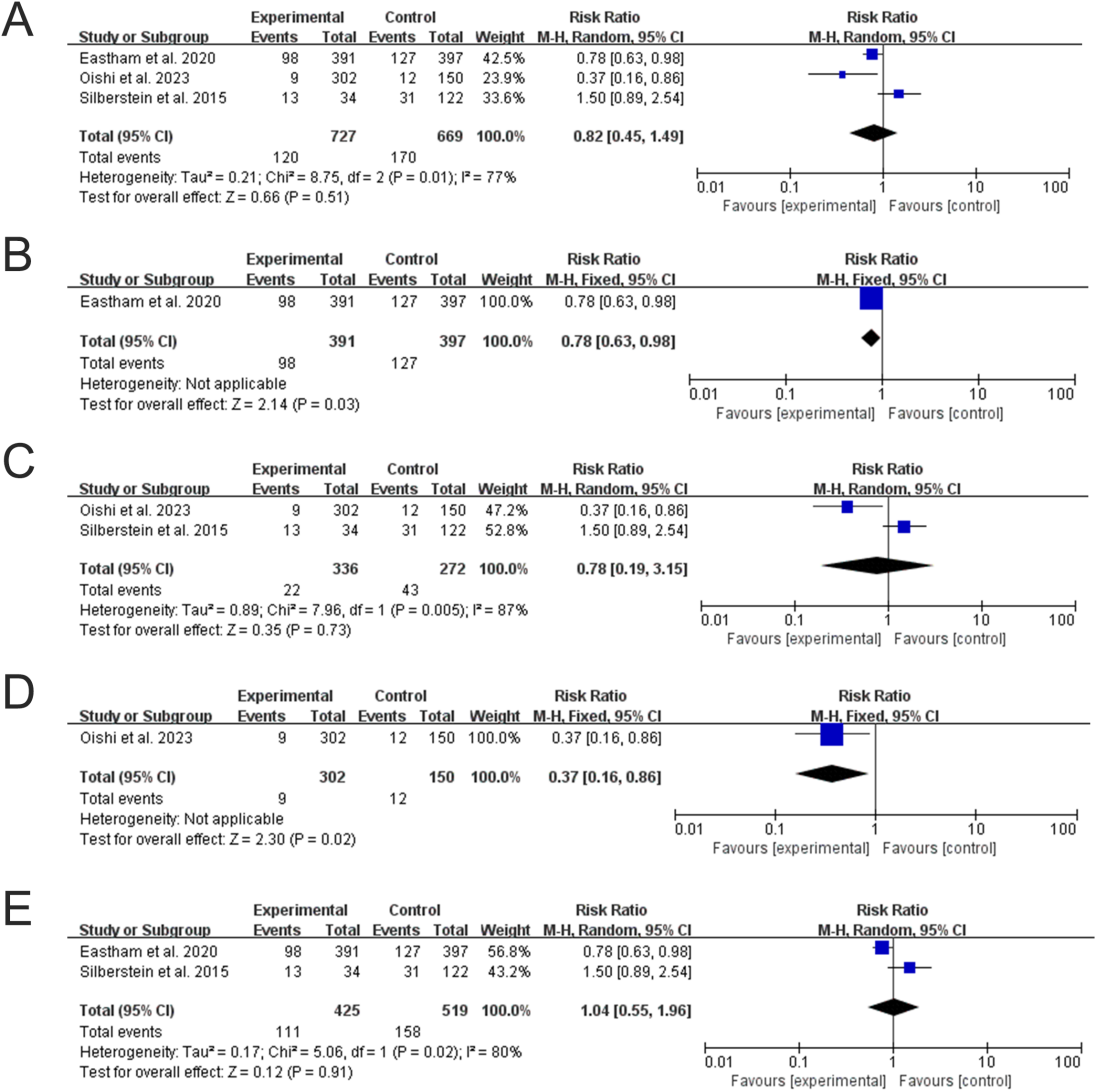
Forest plots for MFS. **(A)** Overall analysis. **(B)** Subgroup analysis of the RCT by Eastham et al. **(C)** Subgroup analysis of non-RCTs. **(D)** Subgroup analysis of Japanese studies. **(E)** Subgroup analysis of US studies.

For metastasis-free survival (MFS), the single large RCT (Eastham et al.) reported a nominally significant benefit in an exploratory analysis favoring NCHT+RP (RR = 0.78, 95% CI: 0.63–0.98; P = 0.03) (**Figure 5B**). In contrast, the two non-randomized studies showed no significant difference (RR = 0.78, 95% CI: 0.19–3.15; P = 0.73) with high heterogeneity (I² = 87%) (**Figure 5C**). The overall pooled analysis, combining all three studies, did not reveal a significant benefit (RR = 0.82, 95% CI: 0.45–1.49; P = 0.51; I² = 77%) (**Figure 5A**), largely due to the imprecision and inconsistency contributed by the observational data. Exploratory regional subgroup analysis suggested a significant benefit in Japanese cohort studies (RR = 0.37, 95% CI: 0.16–0.86; P = 0.02) (**Figure 5D**) but not in US studies (RR = 1.04, 95% CI: 0.55–1.96; P = 0.91) (**Figure 5E**); however, these analyses are based on single studies and should be interpreted with caution.

### Sensitivity analysis

**Figure 6:**
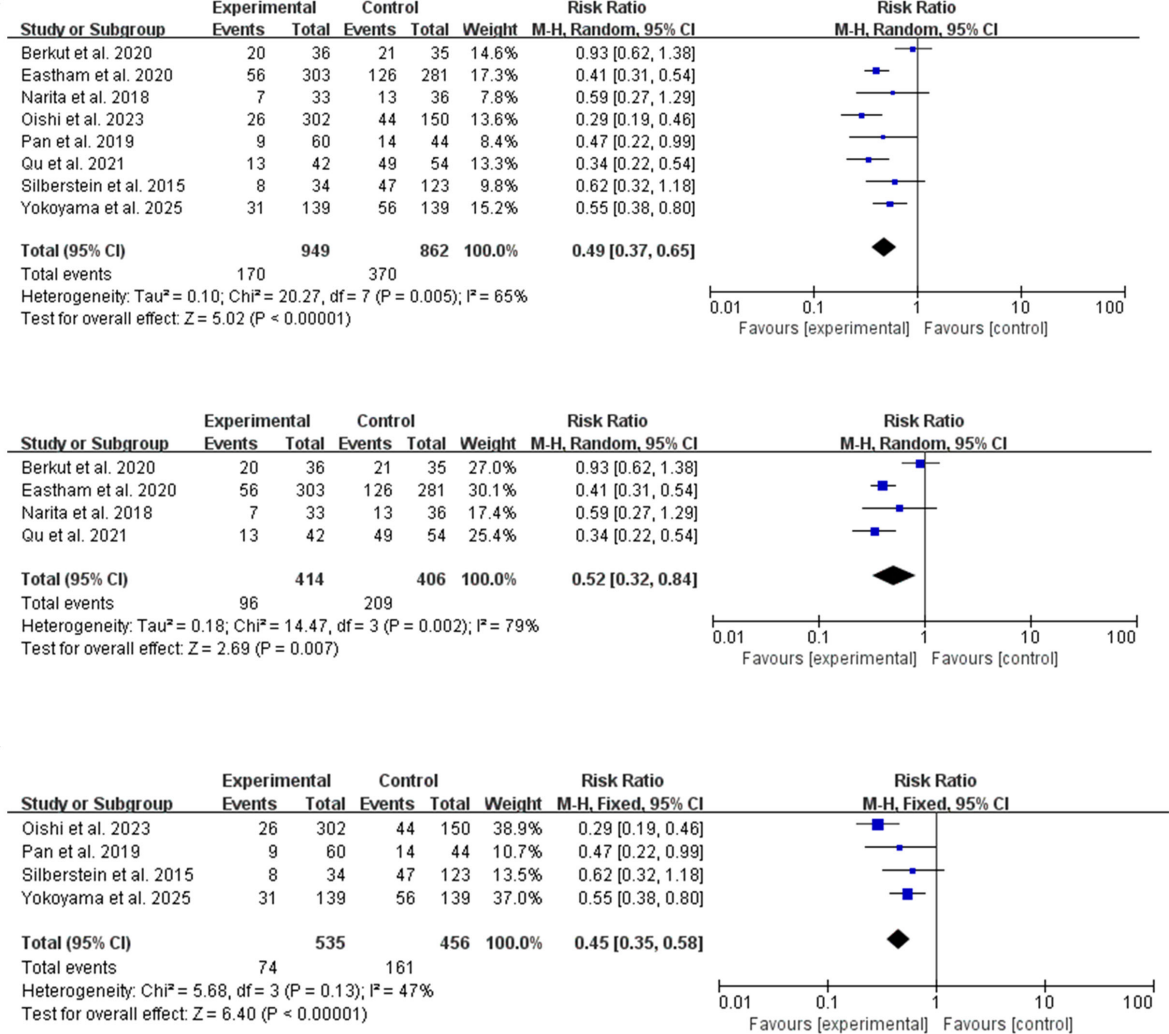
Baujat plot for sensitivity analysis, identifying studies contributing to heterogeneity.

To evaluate how bias risk affects the overall findings, we conducted sensitivity analyses (**Figure 6**). The Baujat plot identified Pan et al., 2019 and Silberstein et al., 2015 as primary contributors to overall heterogeneity. The studies with minimal impact on heterogeneity (Eastham et al., 2020, Yokoyama et al., 2025, Narita et al., 2018) clustered near the origin, indicating robust alignment with the pooled estimate. Notably, Oishi et al., 2023 contributed moderately to heterogeneity.

### Quality assessment and publication bias

**Figure 7:**
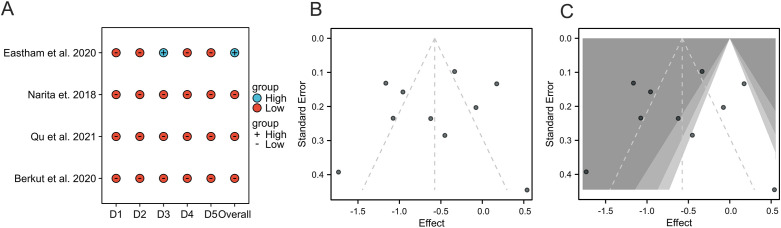
Risk of bias and publication bias assessment. **(A)** Risk of bias summary for included RCTs (Cochrane ROB 2 tool). **(B)** Funnel plot for assessment of publication bias. **(C)** Funnel plot after trim-and-fill analysis.

Among the RCTs, the risk of bias assessment using the ROB 2 tool ranged from “Low” (Berkut et al., Qu et al., Narita et al.) to “High” (Eastham et al.) (**Figure 7A**). The methodological quality of the non-randomized studies, assessed by the NOS, varied (scores 3–9) (**Table 3**). The study by Oishi et al. (score=3) was identified as a major source of heterogeneity. Funnel plots indicated the absence of significant publication bias (**Figure 7B**). The trim-and-fill confirmed robust results, reinforcing the reliability of our results (**Figure 7C**).

### GRADE evidence quality assessment

We used the GRADE (Grading of Recommendations Assessment, Development and Evaluation) framework to assess the certainty of evidence for each primary outcome (**Table 5**). For BPFS and PSM rate, the evidence was rated as Low certainty, downgraded due to serious risk of bias (inclusion of observational studies with potential confounding) and serious inconsistency (substantial statistical heterogeneity). For OS and MFS, the evidence was rated as Very Low certainty, further downgraded for imprecision (wide confidence intervals crossing the line of no effect, and few events in RCTs) and indirectness (variations in chemotherapy regimens and patient populations). This very low certainty indicates that the true effect of NCHT on survival outcomes is likely to be substantially different from the estimated effect.

**Table 5:** Summary of findings table with GRADE ratings for critical outcomes.

Outcomes	GRADE rating	Explanation
Biochemical progression-free survival (BPFS)	Low	Downgraded due to serious risk of bias (inclusion of observational studies with potential confounding) and serious inconsistency (substantial statistical heterogeneity, I² = 90%).
PSM rate	Low	Downgraded due to serious risk of bias and serious inconsistency (moderate to high heterogeneity, I² = 65%).
OS	Very low	Downgraded due to serious risk of bias, serious inconsistency (I² = 61%), imprecision (wide confidence intervals crossing no effect, few events in RCTs), and indirectness (variations in chemotherapy regimens and patient populations).
MFS	Very low	Downgraded due to serious risk of bias, serious inconsistency (I² = 77%), imprecision, and indirectness. The true effect is likely substantially different from the estimate.

BPF, biochemical progression-free survival; PSM, positive surgical margin rate; OS, Overall survival; MFS, Metastasis-free survival.

## Discussion

This meta-analysis demonstrates that NCHT combined with RP offers significant advantages over RP alone in improving BPFS and reducing PSM rates in patients with locally advanced prostate cancer. However, the benefits for MFS were not statistically significant overall, and the improvement in OS was observed primarily in the non-randomized study subgroup. Previous studies have indicated that NCHT may help improve BPFS, but controversy remains regarding its impact on OS and MFS (14). The findings of this meta-analysis provide a nuanced understanding of the efficacy of NCHT. Our results, demonstrating a significant improvement in BPFS and a reduction in PSM rates, are consistent with some previous systematic reviews (24–26), but contrast with others that found no overall survival benefit (29). This updated synthesis incorporates recent trials and highlights the ongoing uncertainty. This consistency underscores the robustness of the local disease control benefit offered by NCHT. The biological plausibility of this benefit is well-supported: the synergistic effect of androgen deprivation and chemotherapy potently induces tumor cell apoptosis and reduces tumor volume, facilitating a more complete surgical resection with negative margins (27). This mechanistic pathway directly translates into the observed reduction in biochemical recurrence, a well-established surrogate endpoint for local treatment efficacy (28).

An important methodological consideration is our decision to pool RCTs and non-randomized studies. While we conducted prespecified subgroup analyses by study design, the overall pooled estimates (especially for OS) are heavily influenced by observational data, which are susceptible to selection bias and confounding. This is clearly illustrated by the OS results: the overall significant effect disappears in the RCT subgroup. Therefore, the main conclusions of this meta-analysis should be based primarily on the RCT evidence, and the non-randomized results should be viewed as hypothesis-generating only.

Despite these positive signals, the largest RCT included in our analysis—CALGB 90203 (Eastham et al.)—reported that neoadjuvant docetaxel-based NCHT did not significantly improve its primary endpoint of 3-year biochemical progression-free survival, and only modest benefits were observed in secondary outcomes (14). This discrepancy highlights the limitations of docetaxel-based regimens and underscores the need for more potent neoadjuvant strategies. Indeed, the field has now shifted toward evaluating ARPI-based combinations, which have shown promising pCR rates in phase II trials (29). Our meta-analysis, therefore, represents a synthesis of the older generation of chemohormonal therapy; its conclusions should not be extrapolated to modern ARPI-containing regimens, which require dedicated randomized trials.

However, our results regarding survival outcomes introduce a critical layer of complexity to the existing literature. The significant improvement in OS in the overall analysis, which was primarily driven by non-randomized studies, stands in contrast to some earlier meta-analyses that found no OS benefit (30). This discrepancy may be attributable to the inclusion of more recent studies with longer follow-up durations or differing patient risk profiles. Importantly, while the RCT subgroup did not demonstrate a statistically significant OS improvement (RR = 0.43, 95% CI: 0.10–1.88; P = 0.26), the point estimate suggesting a substantial 57% reduction in mortality risk highlights a crucial limitation: the current body of RCT evidence is likely underpowered to detect a survival difference. This underscores the imperative for larger, well-designed randomized trials with sufficient statistical power.

Similarly, the neutral overall result for MFS (RR = 0.82, P = 0.51) contrasts with the hypothesis that effective local control should delay metastasis. This divergence from pre-clinical expectations could be explained by several factors. First, the heterogenous use and timing of salvage therapies upon biochemical recurrence across studies may confound the true MFS effect of NCHT (31). Second, it is possible that NCHT, while effective locally, may not adequately eradicate micrometastatic disease present at diagnosis in a subset of high-risk patients, necessitating more effective systemic adjuvant strategies (18, 20, 23). The striking regional heterogeneity we observed—with significant benefit in Japanese cohorts but none in US studies—further complicates the picture. This may not merely reflect differences in clinical practice but could also hint at underlying ethnic variations in tumor biology or pharmacogenomics affecting drug response.

The substantial heterogeneity observed, particularly for BPFS (I² = 90%), warrants careful consideration of its sources and impact on our conclusions. Our sensitivity analysis identified Pan et al. (2019) and Silberstein et al. (2015) as major contributors, likely due to differences in chemotherapy backbone, patient risk profiles, and follow-up duration. In contrast, studies with more homogeneous designs (e.g., Eastham et al., Yokoyama et al.) clustered near the origin in the Baujat plot, indicating greater consistency with the pooled estimate. Despite these exploratory analyses, residual heterogeneity remained unexplained, which contributed to the downgrading of evidence certainty to low or very low for all outcomes in the GRADE assessment. This highlights the need for future studies with standardized protocols and harmonized outcome definitions.

According to the GRADE framework, the certainty of evidence for BPFS and PSM was rated as low, primarily due to serious risk of bias and inconsistency. For OS and MFS, the evidence was rated as very low, further downgraded for imprecision and indirectness. These ratings reflect the limited confidence we can place in the pooled effect estimates and reinforce the exploratory nature of our findings. Clinicians should interpret these results with caution when considering NCHT in routine practice.

Moreover, the results of this study have significant implications for clinical practice, especially in formulating treatment plans for high-risk locally advanced PCa patients. The study suggests that NCHT may serve as an effective treatment option, improving patient prognosis and reducing the risk of biochemical recurrence. These findings not only highlight the potential application value of NCHT in radical treatment but also provide a basis for developing more personalized treatment strategies in the future. However, it is imperative to note that current clinical guidelines (e.g., NCCN) do not recommend neoadjuvant ADT or chemotherapy prior to RP outside of clinical trials. The use of NCHT should remain investigational, and patients should be counseled about the availability of ongoing trials with novel ARPI-based therapies.

Several limitations of this study should be acknowledged. First, methodological constraints include the pooling of RCTs and observational studies, which increases heterogeneity and potential bias. Second, the use of risk ratios rather than hazard ratios for time-to-event outcomes is a significant limitation. Because we used event counts at varying follow-up times, our analyses do not fully incorporate the timing of events, censoring, or differences in follow-up duration between studies. This could bias the effect estimates and reduces the reliability of our survival conclusions. Third, substantial heterogeneity was observed, particularly in the BPFS analysis, likely stemming from variations in study design, patient characteristics, and treatment protocols. Fourth, the number of RCTs was relatively small (n=4) (14, 16, 17, 23), which may limit the robustness and generalizability of the findings. Potential unmeasured confounding factors in the non-randomized studies could also influence the outcomes. Additionally, the geographical concentration of studies (mainly Japan and the US) may limit the broad applicability of our results. Finally, while we performed tests for publication bias, the limited number of included studies (n=10) renders funnel plots and statistical tests such as Egger’s test underpowered to reliably detect bias, which is an inherent limitation of our review.

## Conclusions

In summary, this meta-analysis suggests that for locally advanced PCa, NCHT (docetaxel-based chemohormonal therapy) combined with RP may improve BPFS and reduce PSM rates compared to RP alone, although the certainty of this evidence is low. The apparent OS benefit was not robust in RCT subgroup analyses and may be influenced by confounding in observational studies. According to GRADE, the evidence for survival outcomes is very low certainty, indicating that the true effects may differ substantially from the estimates. Given the modest benefits observed and the evolution of neoadjuvant therapy towards more effective ARPI-based regimens, these findings should be considered hypothesis-generating and primarily of historical context. Well-powered, methodologically rigorous randomized controlled trials evaluating modern combination therapies (e.g., ARPI+ADT) with standardized protocols and long-term follow-up are urgently needed to redefine the role of neoadjuvant treatment in this setting.
