# Supplementary material for: The efficacy of neoadjuvant chemohormonal therapy in combination with radical prostatectomy for locally advanced prostate cancer: a systematic review and meta-analysis
Source: Front Oncol. 2026 Apr 20;16:1693222. doi: 10.3389/fonc.2026.1693222 (PMC13136011; doi:10.3389/fonc.2026.1693222)
Supplement: Supplementary file 2 [file Table2.docx]

Supplement Table 2. Search Strategy

PubMed

| #1 | Neoadjuvant chemohormonal therapy[Title/Abstract] OR Radical prostatectomy[Title/Abstract] OR Locally advanced prostate cancer[Title/Abstract] OR Prostate cancer[Title/Abstract] |
| --- | --- |
| #2 | Prostatectomy[Title/Abstract] OR Surgery[Title/Abstract] OR Chemotherapy[Title/Abstract] OR Hormonal therapy[Title/Abstract] OR Neoadjuvant therapy[Title/Abstract] |
| #3 | Efficacy[Title/Abstract] OR Effectiveness[Title/Abstract] OR Outcomes[Title/Abstract] OR Survival[Title/Abstract] OR Response[Title/Abstract] |
| #4 | #1 AND #2 AND #3 |

Embase

| #1 | Locally advanced prostate cancer' /exp |
| --- | --- |
| #2 | 'Radical prostatectomy' /exp |
| #3 | 'Neoadjuvant chemohormonal therapy' OR 'Neoadjuvant therapy' OR 'Chemotherapy' OR 'Hormonal therapy' OR 'Combination therapy' OR 'Preoperative treatment' |
| #4 | 'Efficacy' OR 'Effectiveness' OR 'Outcome' OR 'Results' OR 'Performance' OR 'Impact' |
| #5 | 'Systematic review' OR 'Review' OR 'Literature review' OR 'Meta-analysis' OR 'Evidence synthesis' OR 'Comprehensive review' |
| #6 | 'Meta-analysis' OR 'Statistical analysis' OR 'Quantitative review' OR 'Data synthesis' OR 'Statistical review' OR 'Combined analysis' |
| #7 | #1 AND #2 AND #3 AND #4 AND #5 AND #6 |

Web of Science

| #1 | TS=((prostate cancer) OR (locally advanced prostate cancer) OR (advanced prostate cancer) OR (prostate carcinoma) OR (prostatic neoplasm)) |
| --- | --- |
| #2 | TS=((neoadjuvant therapy) OR (neoadjuvant treatment) OR (preoperative therapy) OR (preoperative treatment) OR (induction therapy)) |
| #3 | TS=((chemohormonal therapy) OR (chemotherapy and hormone therapy) OR (combined chemotherapy and hormone therapy) OR (chemotherapy with hormonal therapy) OR (hormonal chemotherapy)) |
| #4 | TS=((radical prostatectomy) OR (prostatectomy) OR (surgical removal of prostate) OR (prostate cancer surgery) OR (radical surgical procedure for prostate)) |
| #5 | #1 AND #2 AND #3 AND #4 |

Cochrane

| #1 | MeSH descriptor: [Neoadjuvant Therapy] explode all trees |
| --- | --- |
| #2 | (“Neoadjuvant Chemotherap*” OR “Neoadjuvant Chemotherapy Protocols” OR “Neoadjuvant Treatment” OR “Preoperative Chemotherap*” OR “Induction Chemotherap*”):ti,ab,kw |
| #3 | #1 or #2 |
| #4 | MeSH descriptor: [Prostatic Neoplasms] explode all trees |
| #5 | (Prostate Cancer OR “Prostatic Carcinom*” OR “Prostate Neoplasm” OR “Prostate Tumor” OR “Prostate Adenocarcinom*”):ti,ab,kw |
| #6 | #4 or #5 |
| #7 | MeSH descriptor: [Prostatectomy] explode all trees |
| #8 | (“Radical Prostatectom*” OR “Total Prostatectom*” OR “Surgical Prostatectom*” OR “Prostate Removal Surgery” OR “Prostatectomy Surgery”):ti,ab,kw |
| #9 | #7 or #8 |
| #10 | (“Chemohormonal Therapy” OR “Hormonal Chemotherap*” OR “Hormonal Treatment with Chemotherap*” OR “Combined Hormonal and Chemotherap*” OR “Hormonal Chemotherap* Combination”):ti,ab,kw |
| #11 | (“Locally Advanced Prostate Cancer” OR “Locally Advanced Prostatic Neoplasm” OR “Locally Advanced Prostate Tumor” OR “Locally Advanced Prostate Carcinom*” OR “Locally Advanced Prostate Adenocarcinom*”):ti,ab,kw |
| #12 | (“Efficacy” OR “Effectiveness” OR “Efficiency” OR “Therapeutic Effect” OR “Clinical Effectiveness”):ti,ab,kw |
| #13 | (“Systematic Review” OR “Systematic Literature Review” OR “Comprehensive Review” OR “Meta-Analysis” OR “Evidence Synthesis”):ti,ab,kw |
| #14 | (“Meta-Analysis” OR “Quantitative Review” OR “Statistical Review” OR “Meta-Analytic Study” OR “Meta-Analytic Review”):ti,ab,kw |
| #15 | #3 AND #6 AND #9 AND #10 AND #11 AND #12 AND #13 AND #14 |
